# Testing the proportional hazards assumption in case-cohort analysis

**DOI:** 10.1186/1471-2288-13-88

**Published:** 2013-07-09

**Authors:** Xiaonan Xue, Xianhong Xie, Marc Gunter, Thomas E Rohan, Sylvia Wassertheil-Smoller, Gloria YF Ho, Dominic Cirillo, Herbert Yu, Howard D Strickler

**Affiliations:** 1 Department of Epidemiology and Population Health, Albert Einstein College of Medicine, New York, New York, USA; 2 Department of Epidemiology and Biostatistics, School of Public Health, Imperial College, London, United Kingdom; 3 Department of Internal Medicine, University of Iowa Health Care, Iowa, USA; 4 Cancer Epidemiology Program, University of Hawaii Cancer Center, Honolulu, Hawaii, USA

**Keywords:** Proportional hazards, Schoenfeld residuals, Case-cohort studies, Cox models

## Abstract

**Background:**

Case-cohort studies have become common in epidemiological studies of rare disease, with Cox regression models the principal method used in their analysis. However, no appropriate procedures to assess the assumption of proportional hazards of case-cohort Cox models have been proposed.

**Methods:**

We extended the correlation test based on Schoenfeld residuals, an approach used to evaluate the proportionality of hazards in standard Cox models. Specifically, pseudolikelihood functions were used to define “case-cohort Schoenfeld residuals”, and then the correlation of these residuals with each of three functions of event time (i.e., the event time itself, rank order, Kaplan-Meier estimates) was determined. The performances of the proposed tests were examined using simulation studies. We then applied these methods to data from a previously published case-cohort investigation of the insulin/IGF-axis and colorectal cancer.

**Results:**

Simulation studies showed that each of the three correlation tests accurately detected non-proportionality. Application of the proposed tests to the example case-cohort investigation dataset showed that the Cox proportional hazards assumption was not satisfied for certain exposure variables in that study, an issue we addressed through use of available, alternative analytical approaches.

**Conclusions:**

The proposed correlation tests provide a simple and accurate approach for testing the proportional hazards assumption of Cox models in case-cohort analysis. Evaluation of the proportional hazards assumption is essential since its violation raises questions regarding the validity of Cox model results which, if unrecognized, could result in the publication of erroneous scientific findings.

## Background

Case-cohort design is an efficient and increasingly popular method for conducting prospective epidemiological studies of rare outcomes. Compared with standard longitudinal cohort studies, case-cohort investigations are typically less costly, use less resources, and require less time to conduct, though they entail little loss in statistical power [1-3]. In case-cohort studies, relevant but costly or difficult to obtain information is obtained for only a subset of subjects rather than the entire cohort. Specifically, there are two subject groups: (i) the subcohort - a random sample of all subjects in the cohort with no history of the outcome of interest at baseline, selected without regard to future outcomes. Thus, the subcohort may include some individuals who later become cases; and (ii) the case group - all or random sample of the incident cases of disease, the vast majority of whom will be from outside the subcohort. Furthermore, because the subcohort is a representative sample of the entire cohort without disease at enrollment, it is possible to adopt case-cohort design to study multiple different types of disease outcomes (e.g. multiple types of cancer) using the same subcohort. For example, we present below a recent prospective study of fasting serum insulin levels and the risk of three cancer case groups which involved a single subcohort [4-6].

Case-cohort studies are typically analyzed using Cox proportional hazards (PH) models [7]. Specifically, estimation of the Cox proportional hazards model in a case-cohort analysis is obtained by approximating each instantaneous risk set of the entire cohort included in the partial likelihood function of a standard Cox model by a case-cohort risk set. Several approaches to define a case-cohort risk set have been proposed [1,2,8]. Prentice [1] defined the case-cohort risk set as the following: at the instantaneous moment of an event the case-cohort risk set includes the subject who had the event plus all the subjects in the subcohort who remained in the study but did not have the event at least until that exact time. The Cox-type likelihood function that is conditioned on the case-cohort risk sets is referred to as the pseudolikelihood function [1]. Statistical inferences in case-cohort analyses are then determined based on maximization of this pseudolikelihood function. As the Prentice approach involves an exact pseudolikelihood function and, in large samples the two other well-established approaches [2,8] provide similar results to the Prentice method, this paper focuses exclusively on the latter. Appropriate methods to conduct these analyses are now available in standard software such as SAS and R, which has helped to reduce computational obstacles to the adoption of case-cohort design, and has been a major factor in the growing use of this cost-effective design.

One of the key assumptions of the Cox model is the proportional hazards function assumption. Specifically, the model assumes that each covariate has a multiplicative effect in the hazards function that is constant over time. The PH assumption is often of substantial importance. For example, in a randomized controlled trial, we may wish to know whether one treatment is superior to another uniformly over time or only in the short term. Similarly, in observational studies, it is often important to determine whether a factor is associated with a constantly higher or lower risk of the outcome over time. For example, Bellera et al. [9] showed that the prognostic relevance of tumor grade for breast cancer metastases diminished over time and negative hormone receptor status was associated with an increased risk of metastases early but became protective thereafter.

Many approaches for assessing the PH assumption are available for standard cohort studies, including both graphical methods and statistical tests [10-19]. Graphical approaches are a visual form of screening for non-proportionality which can provide insight into the temporality and the extent of non-proportionality that is otherwise difficult to obtain using statistical methods. Conversely, graphical methods involve a moderate degree of subjectivity in interpretation. Statistical tests typically screen for the lack of fit of a Cox model. Specifically, Grambsch and Therneau [19] have shown that many of these statistical tests are essentially tests of a non-zero slope in generalized linear regression models of the Schoenfeld residuals [11] as a function of event time. As discussed further in the Methods section (below), correlation tests of Schoenfeld residuals and event time (or log of the event), the rank order of event time [13] or Kaplan-Meier survival curve (KM) estimates are among the most frequently used approaches for assessing the PH assumption [14]. These methods are popular since they can be calculated using standard statistical software [20,21] and are easy to interpret.

Methods to assess the PH assumption in case-cohort studies are not well-established. We therefore assessed the possibility of extending the correlation tests of Schoenfeld residuals and event time to case-cohort study analysis. Two questions in particular need to be addressed. First, can the pseudolikelihood function be used to calculate valid Schoenfeld residuals? Second, what function of event time is best to use for this correlation in case-cohort studies? This paper aims to address these issues and empirically evaluate the proposed correlation tests for the assessment of proportionality in case-cohort Cox model analysis. These methods are then applied to a case-cohort study designed to evaluate the associations of insulin and insulin-like growth factor (IGF)-axis protein levels with the development of cancer in postmenopausal women [4].

## Methods

### Testing proportionality using “Schoenfeld Residuals” in case-cohort dataset

Consider a case-cohort study where the subcohort C is a simple random sample of size m selected from the cohort at baseline (m < n). We define an independent counting process {*N*_*i*_(*t*), *t* ≥ 0} for *i* = 1, …, *n* so that *dN*_*i*_(*t*) = 1 if the ith person fails at time t and 0 otherwise and *Y*_*i*_(*t*) is a 0–1 process which indicates whether the ith subject is at risk (i.e., remains in the study) at time t. The intensity function for the {*N*_*i*_(*t*), *t* ≥ 0} is given by

$$Y_{i}\left(t\right)\exp\left(\beta X_{i}\left(t\right)\right)d\Lambda_{0}\left(t\right)$$

Where *X*_*i*_(*t*) is a covariate process of dimension p and *dΛ*_0_(*t*) is an unspecified hazard function and *e*^*β*^ is the hazard ratio (HR) associated with a one unit increase in exposure variable *x*_*i*_(*t*). Let ${\tilde{R}}_{i}\left(t\right)=C\cup \left\{i\right\}$ where the subcohort C is a simple random sample of size m selected from the cohort at baseline with m < n. Then the pseudolikelihood function which is used to determine the statistical inference is defined as follows:

(1) $$\tilde{L}\left(\beta \right)=\prod_{i=1}^{n}\prod_{t}{\left[\frac{\exp\left(\beta X_{i}\left(t\right)\right)}{\sum_{k\in {\tilde{R}}_{i}\left(t\right)}Y_{k}\left(t\right)\exp\left(\beta X_{k}\left(t\right)\right)}\right]}^{dN_{i}\left(t\right)}$$

In a standard Cox model, the estimate of HR can be obtained by maximizing the partial likelihood function. For all the events in the cohort, a separate Schoenfeld residual is defined with respect to each variable in the model. Specifically, for an event in the cohort, its Schoenfeld residual with respect to a variable in the model is defined as the difference between the value of the variable and its mean conditioned upon the risk set at his/her event time (i.e., the subjects who remained in the study without experiencing the event at least until this time). The Schoenfeld residuals are shown to have mean zero under the PH assumption therefore can be used to assess the PH assumption [11].

For purposes of case-cohort analysis, similarly we define the Schoenfeld residual with respect to a given variable for an event occurred at t (either inside or outside the subcohort) as the difference of the covariate value and its mean conditioned upon the case-cohort risk set ${\tilde{R}}_{i}\left(t\right)$. Specifically, for an event occurred at time t with a covariate *X*_*ij*_(*t*) for *j* = 1, …, *p*, the Schoenfeld residual *r*_*ij*_(*t*) is $r_{\mathrm{ij}}\left(t\right)=x_{\mathrm{ij}}\left(t\right)-E\left(x_{\mathrm{ij}}\left(t\right)|{\tilde{R}}_{i}\left(t\right)\right)$ where

$$E\left(x_{\mathrm{ij}}\left(t\right)|{\tilde{R}}_{i}\left(t\right)\right)=\frac{\sum_{k\in {\tilde{R}}_{i}\left(t\right)}Y_{k}\left(t\right)e^{\beta X_{k}\left(t\right)}x_{\mathrm{kj}}\left(t\right)}{\sum_{k\in {\tilde{R}}_{i}\left(t\right)}Y_{k}\left(t\right)e^{\beta X_{k}\left(t\right)}}.$$

Prentice [1] has shown that conditional on event history up to time t, *r*_*ij*_(*t*) has mean 0 under the PH assumption, therefore, it can also be used for assessing proportionality for case-cohort studies. The pseudolikelihood function can be readily constructed using available statistical software by adopting a counting process to describe the event time (i.e., each subject’s time to event process is described by a series of start and stop intervals) [22]. Note that an event that occurs outside the subcohort is assigned a start time immediately before the moment of the event so that this event does not contribute data to any other risk sets. The case-cohort Schoenfeld residuals can then be easily calculated using standard statistical software. We can assess the PH assumption by calculating a Pearson correlation coefficient and its significance for each variable in the model between its Schoenfeld residuals and a function of the corresponding event times, with detection of a significant correlation considered evidence of a violation of the PH assumption. With regards to specific functions of the event time, here we propose to use event time by itself, rank order of the event time and KM estimates. Both rank orders and KM estimates do not assume a parametric form of departure of proportionality, while rank orders are more discrete and depends on only the events that are already occurred and KM estimates depend on the event history (i.e., censorings and events).

Proper adjustment needs to be made to obtain KM estimates for case-cohort data where cases were oversampled. The estimate of increment in cumulative hazard function is a weighted version of Breslow estimator for the full cohort [1], i.e., $d\hat{\Lambda }\left(t\right)=\frac{\sum_{j=1}^{n}dN_{j}\left(t\right)}{n/m\sum_{k\in \tilde{R}\left(t\right)}Y_{k}\left(t\right)}.$ The KM estimate for case-cohort data can therefore be shown as:

$$\hat{S}\left(t\right)={\left[e^{-\sum_{t_{i}\leq t}\frac{\sum_{j=1}^{n}dN_{j}\left(t_{i}\right)}{\sum_{k\in \tilde{R}\left(t_{i}\right)}Y_{k}\left(t_{i}\right)}}\right]}^{m/n}.$$

The R/Splus code to compute for case cohort KM estimates and the three correlation tests is given in the Appendix.

Overall, in case cohort analysis, use of either the event time or rank order of the event time fails to take into consideration of the case-cohort design. The KM estimate has the theoretical advantage since it addresses the oversampling of cases in case-cohort design. These three correlation tests are also commonly used for standard Cox models, for which some simulation has shown that rank order of event time works pretty well [13], there are other situations where the behavior of the different time variables do differ [23]. In below, we used simulation studies to empirically evaluate the performance of these three proposed correlation tests to examine proportionality for a case-cohort Cox model analysis.

### Simulation studies

Several different cohort and subcohort sample sizes were assumed, however, since changes in sample size did not affect the findings, only results for the initial set of assumptions are presented, a sample size of n = 2000 with a random subcohort of m = 500 subjects. A uniform censoring distribution was generated so that the event rate was set to be between 5-10% and all cases outside the subcohort were included in the study. Below, we list additional relevant details related to each of the different scenarios considered in these simulations.

Scenario (1): a Cox model with only one binary variable with 50% exposed. The time to event distribution was varied, as was the hazard ratio. Specifically, we considered the following five situations in which time to event was generated from:

1. A piecewise exponential distribution: the exposed group had hazard rates of 0.1, 0.2, 0.05 and 0.1 at t < 0.3, 0.3 ≤ t < 0.5, 0.5 ≤ t < 0.8 and t ≥ 0.8, respectively and the unexposed group’s hazard rate was proportional to that of the exposed group with a constant HR of 0.5;

2. A piecewise exponential distribution: the exposed group’s hazards rates were 0.1, 0.2, 0.05 and 0.1 while the unexposed group’s rates were 0.05, 0.15, 0.05, 0.15 at t < 0.3, 0.3 ≤ t < 0.5, 0.5 ≤ t < 0.8 and t ≥ 0.8, respectively, so that the HR between exposure and non-exposed groups varied from 2 to 1.3, 1 and 0.7;

3. A Weibull distribution with increasing hazards over time: the scale and shape parameters are (4.0, 1.5) and (4.0, 2.0) for the exposed and unexposed, respectively. For example, when t changes from 0.1 to 0.5, the HR changes from 0.8 to 1.9 at a rate of t^0.5^ (i.e., hazards functions are crossed over);

4. A Weibull distribution with decreasing hazards over time: the scale and shape parameters are (80, 0.3) and (80, 0.6) for the exposed and unexposed, respectively. For example, when t changes from 0.1 to 0.5, the HR changes from 3.7 to 6.0 at a rate of t^0.3^;

5. A Weibull distribution with one constant hazard and another increasing hazards over time: the scale and shape parameters are (4.0, 1.0) and (4.0, 1.5) for the exposed and unexposed, respectively. For example, when t changes from 0.1 to 0.5, the HR changes from 0.9 to 2.1 at a rate of t^0.5^.

Scenario (2): a Cox model with a single continuous covariate generated from a standard normal distribution. We considered the following three situations in which survival times were generated from:

1. A piecewise exponential distribution with hazard rates of 0.1, 0.2, 0.05 and 0.1 at t < 0.3, 0.3 ≤ t < 0.5, 0.5 ≤ t < 0.8 and t ≥ 0.8 and a constant HR of 0.5 per unit increase of the continuous variable;

2. A Weibull distribution with a shape parameter of 1.5, a scale parameter of 4.0, and a constant HR of 1.5;

3. A piecewise exponential distribution with parameters the same as those in Scenario (1.1) but the HR was 0.5, 0.5, 1.0 and 1.0 at the four intervals, respectively.

Scenario (3): a Cox model with more than one covariate. For simplicity, we generated a binary and an independent continuous variable. Since our simulations (shown below) demonstrated that the results are largely robust in relation to the distribution of the survival times, we limited the distribution to a piecewise exponential distribution (scenario (1.1)), a general shape of hazard functions (i.e., non-monotonic). We considered the following situations:

1. Both the binary and the continuous covariates had a constant HR of 0.5;

2. The HR associated with the continuous covariate was constant at 0.5, for the binary variable it was set to be (0.5, 0.5, 1.0, 1.0) for the four intervals, respectively;

3. The HR associated with the binary covariate was constant at 0.5, for the continuous variable it was set to be (0.5, 0.5, 1.0, 1.0) for the four intervals, respectively;

4. Both HRs for the binary and the continuous covariate were varying and at 0.5, 0.5, 1.0, 1.0 for the four intervals, respectively.

A case-cohort analysis was conducted on each simulated data, and then all three proposed correlation tests for proportionality were applied. The simulation was repeated 1000 times and the percent of simulations in which proportionality was rejected (i.e., the percent of times that the p-value from the correlation test was less than 5%) was determined. When the hazards are truly proportional (that is, when the null hypothesis is true), this proportion is the empirical type I error rate of the test; when the hazards are not proportional (that is, when the alternative is true), then this proportion is the empirical power of the test.

### A case-cohort study example

A case-cohort investigation of incident colorectal cancer (ICC) was conducted among non-diabetic subjects enrolled in the Women’s Health Initiative (WHI) Observational Study, a prospective cohort of 93,676 postmenopausal women aged 50 to 79 years who were recruited at 40 clinical centers across the United States between 1993 and 1998 [24]. Fasting baseline serum specimens from all ICC cases (n = 438) and a random subcohort (n = 816) of the WHI observational study subjects were tested for levels of insulin, glucose, total and free insulin-like growth factor (IGF)-I and IGF binding protein (IGFBP)-3. The relation of ICC risk with each of these biomarkers, as well as body mass index (BMI), waist circumference and waist/hip ratio, was assessed in separate multivariate Cox models that employed appropriate methods to account for the case-cohort design. Each primary exposure variable was expressed as quartiles or tertiles based on the distribution of levels in the subcohort. All models were adjusted for a priori established colorectal cancer risk factors including age (categorized as 50 to 54, 55 to 59, 60–64, 65 to 69, 70 to 74 and 75–79 years); smoking status (never, former, and current); race/ethnicity (White, Black, Hispanic, and other); physical activity, assessed as metabolic equivalent tasks per hour per week (categorized as <3.75, 3.75-10, 10–20 and ≥20 METs); waist circumference (categorized as <75.0, 75.0-83.5, 83.5-93.0 and ≥93.0 cm); use of nonsteroidal anti-inflammatory drugs in the preceding year (no/yes), alcohol consumption (none, <3, and ≥3 number of servings/week); and family history of colorectal cancer (no/yes).

The results showed that insulin (HR = 1.21; 95% CI: 1.07-1.36) and waist circumference (HR = 1.23; 95% CI: 1.11-1.35) per quartile increase were significantly associated with ICC risk. However, the proportionality assumption was not examined because of unavailability of appropriate methods for case-cohort studies at that time. In this paper, we used the methods developed to examine the adequacy of the PH assumption for the Cox model that included insulin as the primary exposure variable, and the model that included waist circumference as the primary exposure variable. For the Cox model that contains variable(s) which did not satisfy the assumption, we used a stratified Cox model [22] or an additive hazards model for case-cohort datasets [25,26]. For the stratified Cox model, the method for obtaining a robust variance estimator for confounder-stratified case-cohort studies was used. In an additive model, the hazard function is defined as

$$\lambda \left(t\right)=\lambda_{0}\left(t\right)+\gamma X_{i}\left(t\right)$$

in which the covariate *X*_*i*_(*t*) acts additively on the hazards function and *γ* has the interpretation as the absolute increase in instantaneous event rate per unit increase in the variable. Therefore, the PH assumption is no longer needed in this model. Goodness of fit for the additive hazard model was examined [27].

All the analyses were conducted using R (R 2.12.2, 2011, The R Foundation for Statistical Computing).

## Results

### Simulation study

**Figure 1:**
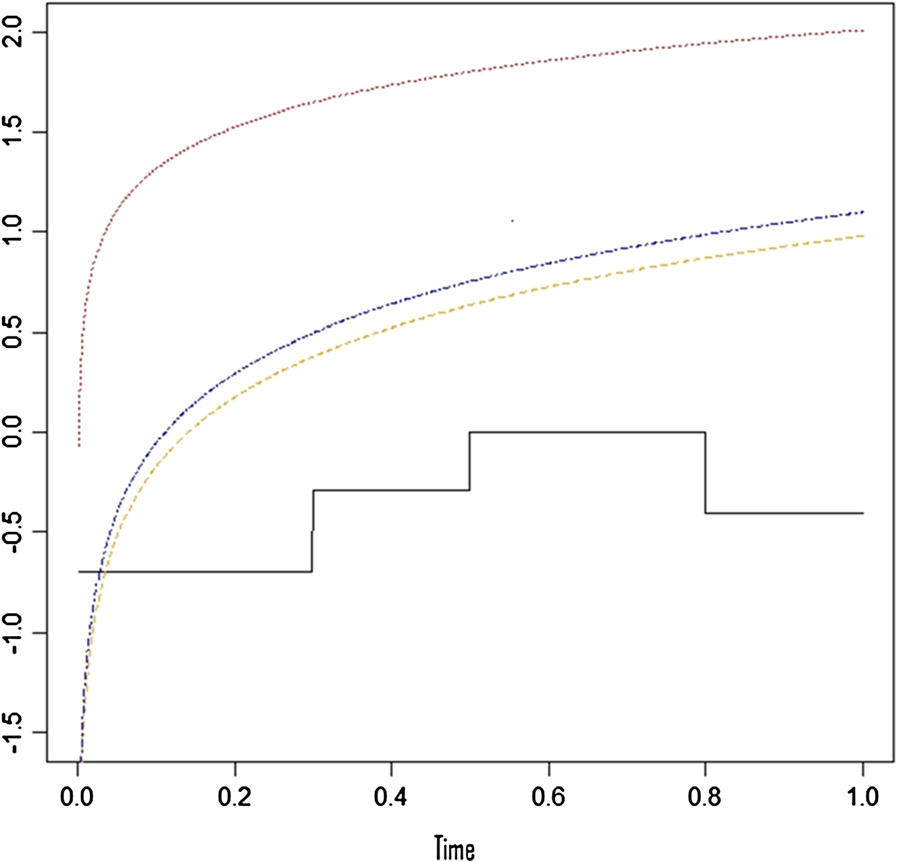
**Log of ratio of hazards functions between two categories of a binary exposure variable under various of simulated non PH scenarios of non PH listed in Table**1**.** Black line represents non-monotonic hazard functions and non PH; orange line represents increasing hazards and non PH; red line represents decreasing hazards and non PH; blue line represents one constant and one increasing hazards and non PH.

Table 1 shows that with a single binary variable, all three correlation tests have a similar level of type I error, though the correlation tests based on the event time and the rank order were slightly higher than the correlation test based on the KM estimates (5.9%, 5.6% and 5.4%, respectively). In most scenarios, all three tests provided similar results except when both hazard functions are declining but not proportional, where the correlation test based on event time had the lowest power (80.5% vs 93.1% and 92.8%). Figure 1 indicates that the log of hazard ratio under the fourth scenario (i.e., declining hazards and non-proportional) can be least approximated by a linear function of survival time among the four scenarios. This explains why the correlation with time has the lowest power here as the other two tests are more robust to the shape of the hazard ratio over time. Although the KM estimate has the advantage of addressing the case-cohort design and being a more smoothed curve than the rank order, it does not seem advantageous over rank in detecting non-proportionality. In fact, the two tests have very close results. Same as the findings from standard Cox model, the performance of the correlation test depends on the true form of the time-dependent hazard ratio. Therefore, the decision on which time variable to use is typically case by case, largely depending on the researchers’ understanding of the true exposure and disease association.

**Table 1:** **Proportion of simulations** (**out of 1000**) **with P**-**values** < **0**.**05 for a single variable**

**Variable type**	**Shape of hazards functions & proportionality**	**Corr with time**	**Corr with rank**	**Corr with KM estimate**
Binary	Non-monotonic proportional	0.059	0.056	0.054
	Non-monotonic non-proportional	0.874	0.877	0.857
	Increasing non-proportional	0.445	0.409	0.407
	Decreasing non-proportional	0.805	0.931	0.928
	One constant one increasing non-proportional	0.657	0.666	0.646
Continuous	Non-monotonic proportional	0.052	0.059	0.060
	Increasing proportional	0.041	0.045	0.042
	Non-monotonic non-proportional	0.997	0.996	0.995

Note: Abbreviation: *Corr* correlation, *KM* Kaplan-Meier Survival Curve.

The full-cohort sample size for all models was 2000; group 1 and group 2 had equal sample size; and the subcohort sample size was 500 using simple random sampling.

Table 1 also shows that with a continuous variable, correlation with event time had slightly better type I error (5.2% vs 5.9% and 6.0%). In the other scenarios, all three tests provided similar results. Table 2 shows that the tests distinguished the variables that satisfied the proportionality assumption and the variables that did not when they were in the same model. In summary, the three tests performed equally well.

**Table 2:** **Proportion of simulations** (**out of 1000**) **with P**-**values** < **0**.**05 for one continuous variable and one binary variable**

**Proportionality**	**Variable**	**Corr with time**	**Corr with rank**	**Corr with KM estimate**
Proportionality for both variables	Continuous	0.059	0.056	0.056
	Binary	0.042	0.043	0.045
Proportionality for the continuous but not the binary variable	Continuous	0.047	0.048	0.045
	Binary	0.646	0.641	0.647
Proportionality for the binary but not the continuous variable	Continuous	0.979	0.971	0.967
	Binary	0.043	0.045	0.045
Non-proportionality for both variables	Continuous	0.994	0.991	0.989
	Binary	0.597	0.586	0.580

Note: Non-monotonic hazards function was assumed.

### The case-cohort study example

**Figure 2:**
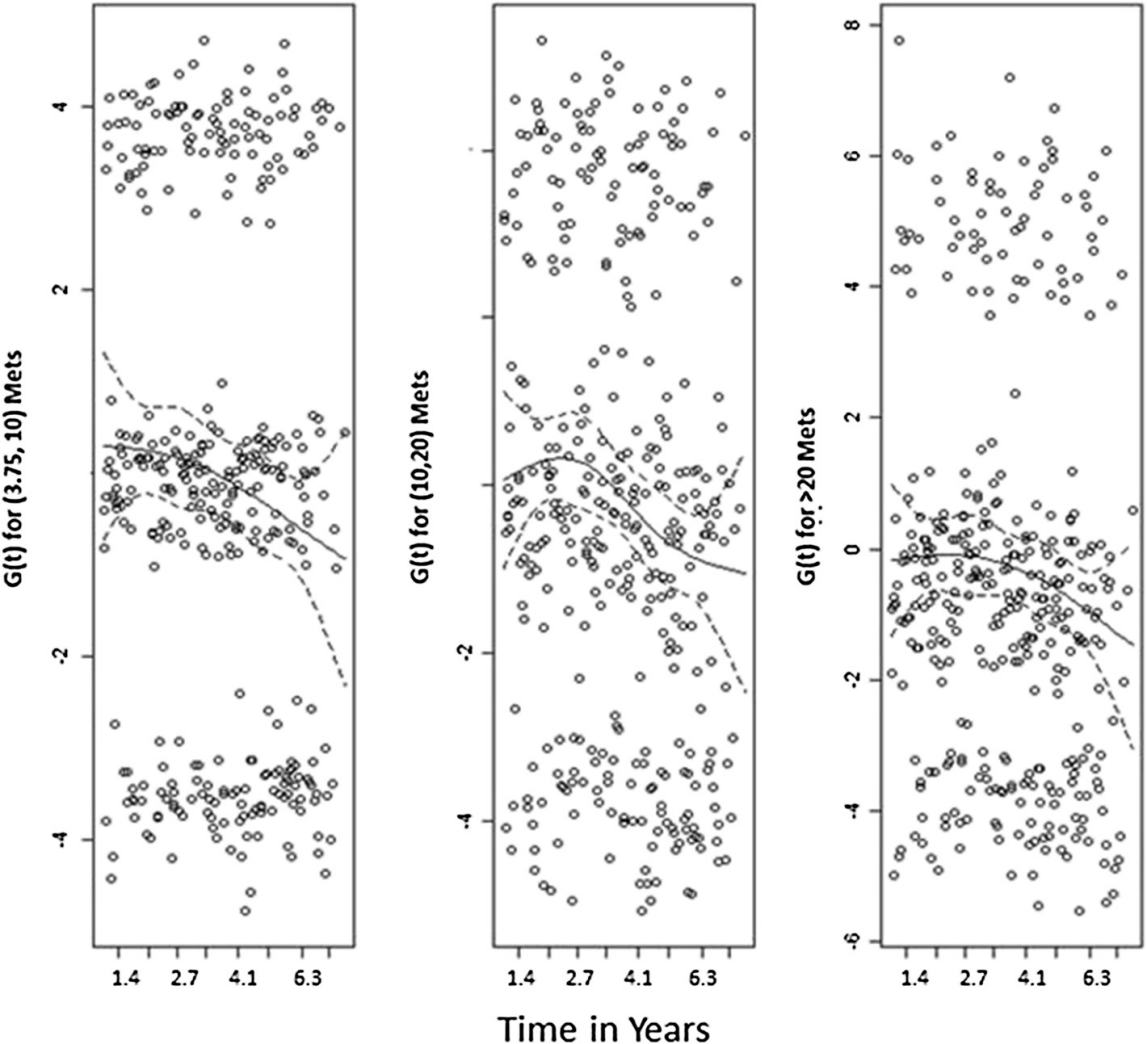
**Schoenfeld residuals for physical activity in the multivariate Cox model for the example case-cohort study of colorectal cancer risk, using insulin levels as the primary exposure variable.** For variable definitions, please see foot note for Table 3. The solid smooth line is the estimated lowess smoothed curve of G(t), i.e., the time-dependent departure of proportionality, and the dotted lines are the estimated confidence bands.

In the Cox model that included insulin as the primary exposure variable the variable “physical activity” failed to satisfy the PH assumption (Table 3), i.e., the hazards function for 10–20 METs of physical activity was not proportional to the reference level. We then graphically examined how the departure from proportionality had occurred. A smoothed curve of the scaled Schoenfeld residuals for physical activity served as an estimate of the time-dependent departure from proportionality, termed G(t), with *β* + *G*(*t*) representing the association between ICC and physical activity if the relationship varied over time. A flat curve close to 0 is expected if the PH assumption is satisfied. Given $\hat{\beta }=-0.3177$, Figure 2 shows that the relation of ICC with 10–20 Mets was weaker during the first three years than in subsequent years. To address this source of non-proportionality, we stratified the analysis by physical activity stratum. In this new model, insulin remained significant with an HR of 1.18 (CI: 1.04, 1.34) per quartile increase in insulin level, an estimate very close to but smaller than the previous estimate. In other words, the estimate was more conservative after the adjustment for non-proportionality.

**Table 3:** **Assessment of proportional hazards for each variable in the multivariate cox model for the example case**-**cohort study of colorectal cancer risk**, **using insulin levels as the primary exposure variable**

**Covariates in the model**	**Corr with event time**	**Corr with rank of time**	**Corr with KM estimates**
Insulin	0.154	0.176	0.164
Mets for physical activity: (0,3.75)-ref			
(3.75,10)	0.389	0.384	0.397
(10,20)	0.045	0.040	0.044
> = 20	0.629	0.686	0.645
Ethnic: white -ref			
Black	0.305	0.304	0.294
Hispanic	0.167	0.166	0.176
Others	0.820	0.586	0.704
Family history of colorectal cancer	0.628	0.598	0.624
History of colonoscopy	0.099	0.076	0.089
Smoking: none-ref			
Former	0.962	0.858	0.887
Current	0.613	0.594	0.598
Alcohol consumption: none-ref			
(0,3)	0.491	0.326	0.411
> = 3	0.219	0.256	0.255
NSAID	0.059	0.105	0.075
Age group continuous	0.322	0.181	0.261

Note: Abbreviation: *Mets* metabolic equivalent tasks per hour per week, *NSAID* use of nonsteroidal anti-inflammatory drugs in the preceding year.

Multivariate Cox model was applied to the WHI case-cohort study with insulin as the primary exposure variable, categorized as quartile groups based on the distribution in the subcohort and then treated as a linear trend. The model included age group categorized as (a) 50 to 54 years of age (referent), (b) 55 to 59, (c) 60–64, (d) 65 to 69, (e) 70 to 74, (f) 75–79.

**Figure 3:**
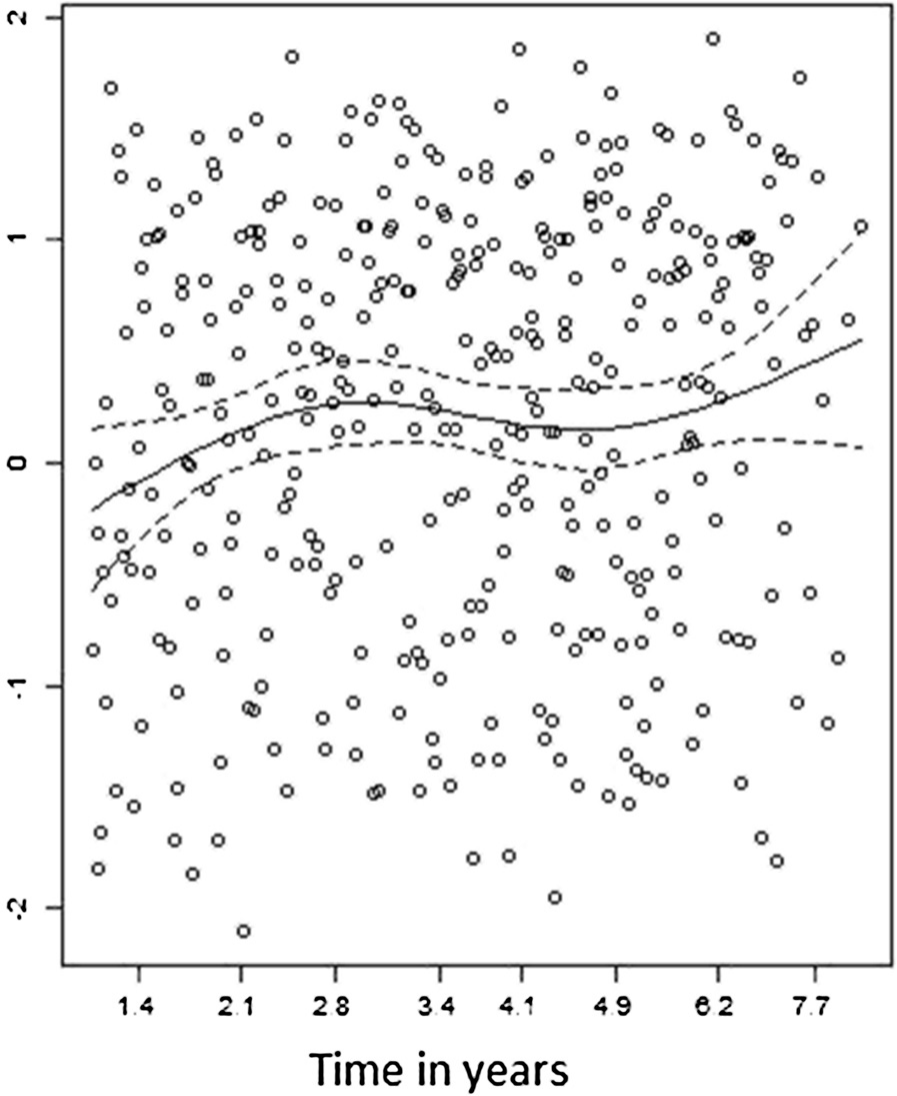
Schoenfeld residuals for waist circumference in the multivariate Cox model for the example case-cohort study of colorectal cancer risk, using in waist circumference levels as the primary exposure variable.

Table 4 shows results for the Cox model that included waist circumference as the primary exposure variable. For simplicity, only the variables that violated the PH assumption are presented, namely, waist circumference and physical activity. The residual plot is shown in Figure 3 and suggests that relation of ICC with waist circumference $\hat{\beta }+\hat{G}\left(t\right)$ ($\hat{\beta }=0.2011$) was comparatively weak during the first two years of follow-up and then increased over time. Therefore, the previous result that a greater waist circumference quartile is associated with a constant 23% higher ICC risk over time (HR = 1.23 per quartile level increase) can be misleading. Because waist circumference is the primary exposure variable of interest it is not possible to stratify the analysis according to this variable since it would prevent estimation of its coefficient. We therefore used an additive model for case-cohort studies. In this new model, waist circumference remained significantly associated with ICC risk and had an effect estimate of 1.41*10^-4^ (CI: 0.46*10^-4^,2.36*10^-4^), suggesting that there will on average be 1.4 extra ICC cases per quartile increase in waist circumference for every 1,000 subjects during 10 years of follow-up. The goodness of fit for the additive model was examined and no lack of fit was indicated.

**Table 4:** **Assessment of proportional hazards for each variable in the multivariate Cox model for the example case**-**cohort study of colorectal cancer risk**, **using waist circumference as the primary variable**

**Covariates in the model**	**Corr with event time**	**Corr with rank of time**	**Corr with KM estimates**
Waist	0.030	0.024	0.026
METs of physical activity: (3.75,10)	0.365	0.361	0.373
(10,20)	0.055	0.049	0.053
> = 20	0.629	0.688	0.645

*Note: Model description is the same as above except that in this model waist circumference is the primary exposure variable. For simplicity, we only listed the variables that do not satisfy proportionality assumption in the multivariate Cox model.

## Discussion

In keeping with its name, the proportionality of the hazards is a critical assumption of Cox proportional hazards analysis. Violation of the PH assumption can raise questions regarding the validity of the model, and possibly lead to misleading and erroneous scientific findings. Because of the importance of assessing the PH assumption, several statistical tests have been proposed for detecting departure from PH in Cox models in the analysis of traditional cohort studies. However, no similar appropriate methods have previously been proposed to assess PH in Cox models of case-cohort data.

In this paper, we extended the correlation tests based on Schoenfeld residuals that are commonly employed to assess PH in standard Cox models so that they might be utilized in case-cohort Cox models. Specifically, we for the first time defined Schoenfeld residuals based on the exact pseudolikelihood function reported by Prentice for the analysis of case-cohort data, then proposed a correlation test between these Schoenfeld residuals and three functions of event time, namely, event time itself, the rank order of the event times, and K-M estimates. We focused on these correlation tests because the interpretation of the results is straightforward and they can be easily implemented using existing software. The simulation studies showed that each of the three correlation tests performed well across multiple simulated scenarios; although, which specific test performed best varied slightly depending on a given scenario. As it is easy to conduct these tests, we recommend that all three tests be performed and that, as a conservative approach, the lowest p-value among these tests be reported. Any indication of violation should be further examined using graphical approaches, which not only overcome the potential problem of multiple testing but also provide more insight regarding the nature of the non-proportionality and how to modify the Cox model to best fit the data.

As mentioned in the introduction section, score tests based on scaled Schoenfeld residuals are also commonly used for standard Cox models [19]. However, because cases outside the subcohort induce non-nesting of the so-called sigma fields, the asymptotic distribution theory for the pseudo-likelihood estimators breaks down so that adjustments were made on the asymptotic variance [2]. More research is needed to extend the score test on scaled Schoenfeld residuals to case-cohort data.

Modifications of the Cox model to address non-proportionality for standard cohort studies include stratification, use of time-dependent covariates, the inclusion of an interaction term between exposure and a function of time, as well as the use of additive hazards models. The first three approaches can be easily implemented for case-cohort studies. However, the use of additive models for case-cohort studies needs to be further developed to allow additive effects to vary by time and to allow multiplicative and additive combined models [28].

In case cohort designs, it is often useful to select subcohort members using an exposure-based stratified random sampling in order to gain statistical efficiency [29]. For example, in a study of epithelial growth factor receptor (EGFR) gene repeat length polymorphism and radiation exposure joint effects on lung cancer risk, an alternative sampling approach to the simple random sampling method was used to randomly select subcohort members within each stratum of their radiation exposure [30]. Generalization of the currently proposed methods for assessing PH, so that they can be used in case-cohort design with stratified sampling will be an important topic for future research.

Although not our main focus, this study also served to further show that insulin is significantly associated with a higher risk of ICC consistently over time, although the estimate of the insulin HR was somewhat more conservative than previously reported following adjustment for the non-proportionality of one of the covariates in the model. We also demonstrated that increased waist circumference does not have a uniform association with ICC risk over time. We then estimated the association of waist circumference on ICC using an additive model.

## Conclusions

To our knowledge, the current paper is the first to report appropriate methods to assess PH in Cox models using data from case-cohort studies. These methods can be easily conducted using standard statistical software, and it is hoped that they will be adopted in the analysis of case-cohort studies to improve the validity of their reported results.

## Appendix

After defining the counting process to describe event time for case cohort data with a subcohort of size m and a cohort of size n, the following R/Splus program can be used to compute the three proposed correlation tests:

# compute schoenfeld residuals

coxfit < − coxph(Surv(start,stop,event) ~ z, data = ccdat, method = "breslow", robust = T)

sresid < − resid(coxfit, type = "schoenfeld")

# compute KM estimate

sfit < − survfit(Surv(start,stop,event) ~ 1, data = ccdat)

sest < − sfit$surv[sfit$n.event > 0]

ecnt < − sfit$n.event[sfit$n.event > 0]

km < − rep(sest^(m/n), ecnt)

# correlation test with event time

cor.test(sort(ccdat$stop[ccdat$event==1]),sresid,method = "pearson")

# correlation test with rank order of event time cor.test(rank(sort(ccdat$stop[ccdat$event==1])),sresid,method = "pearson”)

# correlation test with KM estimates

cor.test(km,sresid,method = "pearson")$p.value

## Abbreviations

IGF: Insulin/insulin-like growth factor; PH: Proportional hazards; HR: Hazards ratio; CI: Confidence interval.

## Competing interests

The authors declare that they have no competing interests.

## Authors’ contributions

XX (Xue) made contributions to every aspect of the study including method development, design of simulations, data analysis, drafting and reviewing the manuscript; XX (Xie) made contributions to method development, programming, conduction of simulations and reviewing the article; MG made contributions to acquisition of data and interpretation of the data analysis results; TR made contributions to interpretation of the results and reviewed the manuscript critically for important intellectual content; SS made contributions to acquisition of the data and reviewed the manuscript critically for important intellectual content; GH made contributions to the conception and the design and review of the manuscript critically for important intellectual content; DC made contributions to the conception and the design and reviewed the manuscript critically for important intellectual content; HY contributed to data acquisition and reviewed the manuscript critically for important intellectual content; HS made substantial contributions to the conception and design and the analysis and interpretation of data, helped to draft the manuscript and critically reviewed the manuscript in great detail. All authors read and approved the final manuscript.

## Authors’ information

XX (Xue) is a professor of Biostatistics, XX (Xie) is research associate, TR, SW, GH and HD are professors of Epidemiology in the Department of Epidemiology and Population Health, Albert Einstein College of Medicine, New York, New York, USA; MG is a reader in Epidemiology, Department of Epidemiology and Biostatistics, School of Public Health, Imperial College, London, United Kingdom; DC is an associate in the Department of Internal Medicine, University of Iowa Health Care, Iowa, USA and HY is a professor in epidemiology and director of Cancer Epidemiology Program, University of Hawaii Cancer Center, Hawaii, USA

## Pre-publication history

The pre-publication history for this paper can be accessed here:

http://www.biomedcentral.com/1471-2288/13/88/prepub

## References

[B1] Prentice RL A case-cohort design for epidemiologic cohort studies and disease prevention trials Biometrika 1986 73 1 11 10.1093/biomet/73.1.1

[B2] Self SG Prentice RL Asymptotic distribution theory and efficiency results for case-cohort studies Ann Stat 1988 16 64 81 10.1214/aos/1176350691

[B3] Xue X Hoover DR Statistical methods in cancer epidemiological studies Cancer Epidemiology Methods Mol Biol 2009 471 239 272 10.1007/978-1-59745-416-2_13 19109784

[B4] Gunter MJ Hoover DR Yu H Wassertheil-Smoller S Rohan TE Manson JE Howard BV Wylie-Rosett J Anderson GL Ho GYF Kaplan RC Li J Xue X Harris TG Burk RD Strickler HD Insulin, insulin-like growth factor-I, endogenous estradiol, and risk of colorectal cancer in postmenopausal women Cancer Res 2008 68 1 329 337 10.1158/0008-5472.CAN-07-2946 18172327 PMC4225702

[B5] Gunter MJ Hoover DR Yu H Wassertheil-Smoller S Manson JE Li J Harris TG Rohan TE Xue X Ho Y Einstein M Kaplan RC Burk RD Wylie-Rosett J Pollak MN Anderson G Howard BV Strickler HD A prospective evaluation of insulin and insulin-like growth factor-I as risk factors for endometrial cancer Cancer Epidemiol Biomarkers Prev 2008 17 4 1 9 18398032 10.1158/1055-9965.EPI-07-2686 PMC3090086

[B6] Gunter MJ Hoover DR Yu H Wassertheil-Smoller S Rohan TE Manson JE Li J Ho Y Xue X Anderson GL Kaplan RC Harris TG Howard BV Wylie-Rosett J Burk RD Strickler HD Insulin, insulin-like growth factor-I, and risk of breast cancer in postmenopausal women JNCI 2009 101 1 48 60 19116382 10.1093/jnci/djn415 PMC2639294

[B7] Cox DR Regression models and life tables (with discussion) J R Stat Soc B 1972 34 187 220

[B8] Barlow WE Robust variance estimation for the case-cohort design Biometrics 1994 50 1064 1072 10.2307/2533444 7786988

[B9] Bellera CA MacGrogan G Debled M de Lara CT Brouste V Mathoulin-Pelissier S Variables with time-varying effects and the cox model: some statistical concepts illustrated with a prognostic factor study in breast cancer BMC Med Res Methodol 2010 10 20 10.1186/1471-2288-10-20 20233435 PMC2846954

[B10] Andersen PK Testing goodness of fit of cox's regression and life model Biometrics 1982 38 67 77 10.2307/2530289

[B11] Schoenfeld D Partial residuals for the proportional hazards regression model Biometrika 1982 69 239 241 10.1093/biomet/69.1.239

[B12] Wei LJ Testing goodness of fit for proportional hazards model with censored observations J Am Stat Assoc 1984 79 649 652 10.1080/01621459.1984.10478092

[B13] Harrell FE Lee KL Verifying Assumptions of the Cox Proportional Hazards Model. Proceedings of the Eleventh Annual. SAS User's Group International Conference 1986 Cary, N.C.: SAS Institute, Inc. 823 828

[B14] Hosmer DW Jr Lemeshow S May S Applied Survival Analysis: Regression Modeling of Time to Event Data 2008 2

[B15] Barlow WE Prentice RL Residuals for relative risk regression Biometrika 1988 75 65 74 10.1093/biomet/75.1.65

[B16] Therneau TM Grambsch PM Fleming TR Martingale-based residuals for survival models Biometrika 1990 77 147 160 10.1093/biomet/77.1.147

[B17] Gray RJ Some diagnostic methods for cox regression models through hazard smoothing Biometrics 1990 46 93 102 10.2307/2531633 2190638

[B18] Lin DY Wei LJ Ying Z Checking the cox model with cummulative sums of martingale-based residuals Biometrika 1993 80 557 572 10.1093/biomet/80.3.557

[B19] Grambsch PM Therneau TM Proportional hazards tests and diagnostics based on weighted residuals Biometrika 1994 81 3 515 526 10.1093/biomet/81.3.515

[B20] Chen X Score Test of Proportionality Assumption for Cox Models 2008 Los Angeles, CA: Statistical Consulting Group UCLA http://www.ats.ucla.edu/stat/

[B21] Therneau T R Package ‘survival’ 2012 http://r-forge.r-project.org23837715

[B22] Langholz B Jiao J Computational methods for case-cohort studies Comput Stat Data Anal 2007 51 3737 3748 10.1016/j.csda.2006.12.028

[B23] Ng’andu NH A comparison of test statistics for assessing the proportional hazards assumption of Cox’s model 1994 Doctor Dissertation at Department of Biostatistics, University of North Carolina, Institute of Statistics, Mimeo Series No. 2141T

[B24] Design of the Women’s Health Initiative Clinical Trial and Observational Study The women’s health initiative study group Control Clin Trials 1998 19 61 109 9492970 10.1016/s0197-2456(97)00078-0

[B25] Aalen OO Klonecki W, Kozek A, Rosinski J A model for non-parametric regression analysis of counting processes Lecture Notes in Statistics-2: Mathematical statistics and Probability Theory 1980 New York, NY: Springer-Verlag 1 25

[B26] Kulich M Lin DY Additive hazards regression for case-cohort studies Biometrika 2000 87 1 73 87 10.1093/biomet/87.1.73

[B27] Xie X Strickler HD Xue X Additive hazard regression models: an application to the natural history of human papillomavirus Comput Math Methods Med 2013 In press 10.1155/2013/796270 PMC3569891 23424606

[B28] Martinussen T Scheike TH Dynamic regression models for survival data 2005 New York, NY: Springer

[B29] Borgan O Langholz B Samuelsen SO Goldstein L Pogoda J **Exposure stratified case-cohort designs** Lifetime Data Anal 2000 6 1 39 10.1023/A:1009661900674 10763560

[B30] Cologne J Preston DL Kazue I Misumi M Yoshida K Hayashi T Nakachi K **Conventional case–cohort design and analysis for studies of interaction** Int J Epidemiol 2012 41 1174 1186 10.1093/ije/dys102 22815332

